# Supplementary material for: Mycobacterium tuberculosis FtsB and PerM interact via a C-terminal helix in FtsB to modulate cell division
Source: J Bacteriol. 2025 Mar 26;207(4):e00444-24. doi: 10.1128/jb.00444-24 (PMC12004960; doi:10.1128/jb.00444-24)
Supplement: Supplemental figures — Fig. S1 to S4. [file jb.00444-24-s0001.pdf]

Supplementary Figures

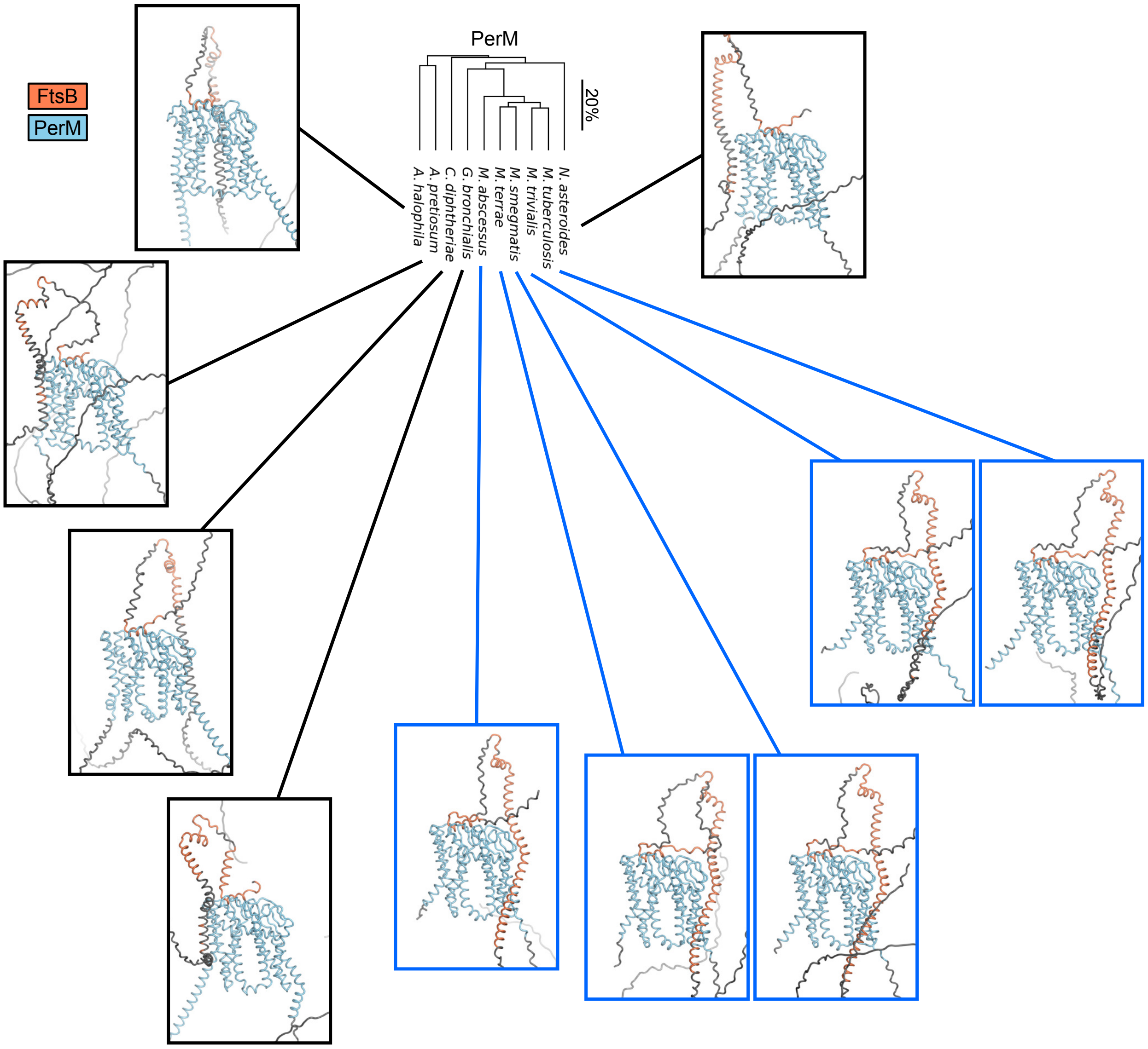

Fig. S1 The PerM phylogenetic tree from Fig. 1 is reproduced and compared to PerM-FtsB complexes predicted from full-length sequences for the same species. The predicted orientation of $FtsB^{TM}$ relative to PerM observed for Mtb is only observed for more closely related species (blue lines). However, $FtsB^{H}$ interaction with PerM is predicted for all species. Residues with $pLDDT < 50$ are colored gray.

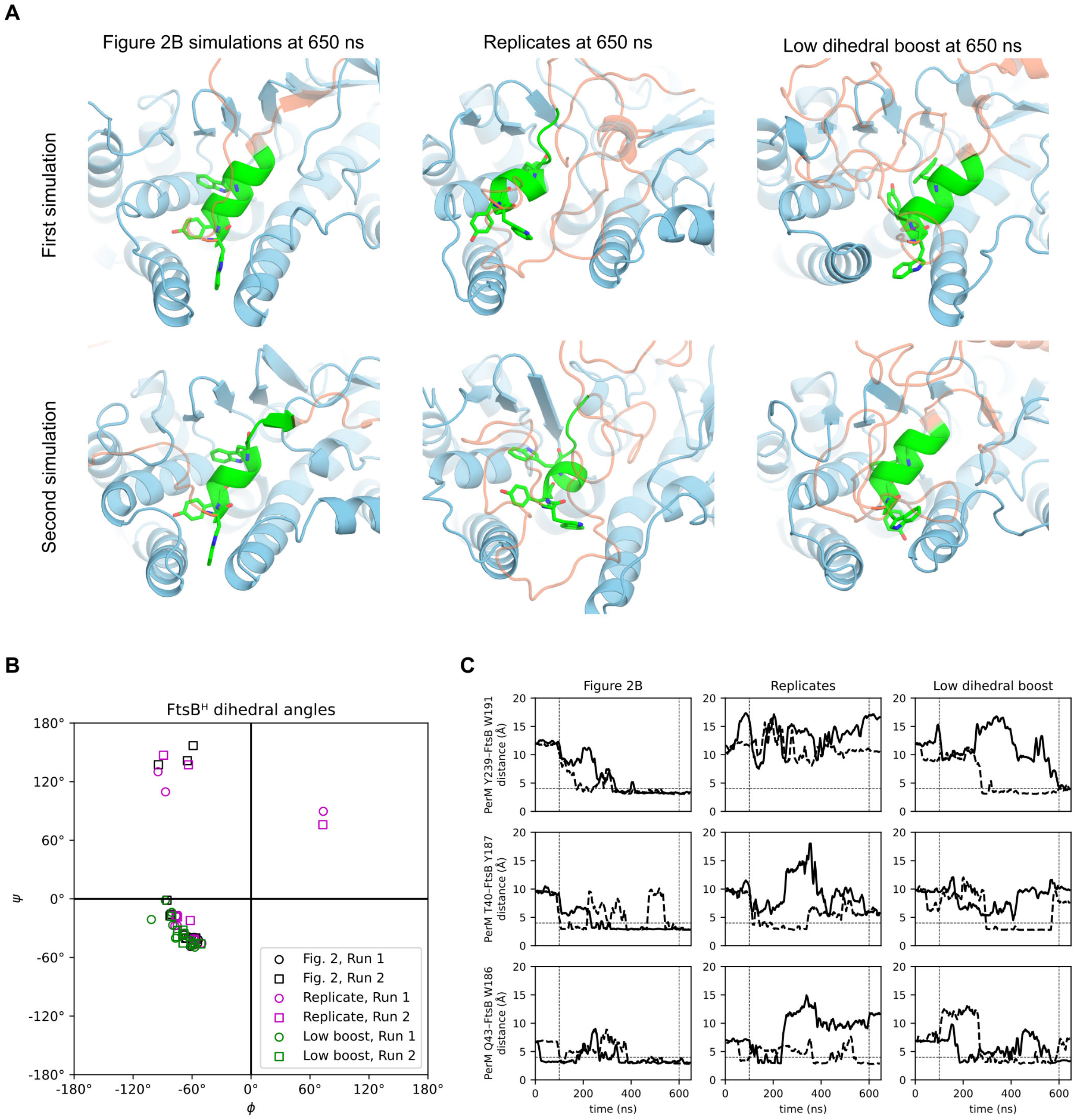

Fig. S2 (**A**) Left: conformers at 650 ns for the PerM-FtsB aMD simulations described in the main text; FtsB^{H} residues are shown in green with a licorice representation for residues W186, Y187, and W191. Other FtsB residues are transparent orange and PerM is blue. FtsB^{H} secondary structure is disrupted in the second simulation. Middle: final conformers in two additional 650 ns replicate simulations. FtsB^{H} secondary structure is disrupted in both simulations. Right: final conformers in two 650 ns simulations in which λ value used to define dihedral boost parameters is reduced from 0.3 to 0.15. FtsB^{H} secondary structure is maintained in both simulations. (**B**) Ramachandran plot for FtsB^{H} residues in 650 ns conformers shows that α-helical dihedral angles are maintained for the first simulation in Fig. 2 and for both low boost simulations, but lost for residues in other simulations. (**C**) Left: reproduction of Fig. 2B plotted through 650 ns; a horizontal line is added at 4 Å. Middle: In replicate simulations that lose FtsB^{H} α-helical secondary structure, hydrogen bonds identified at the PerM-FtsB interface rarely form and a bond never forms between PerM Y239 and FtsB W191. Right: These hydrogen bonds form more frequently when the dihedral boost is reduced.

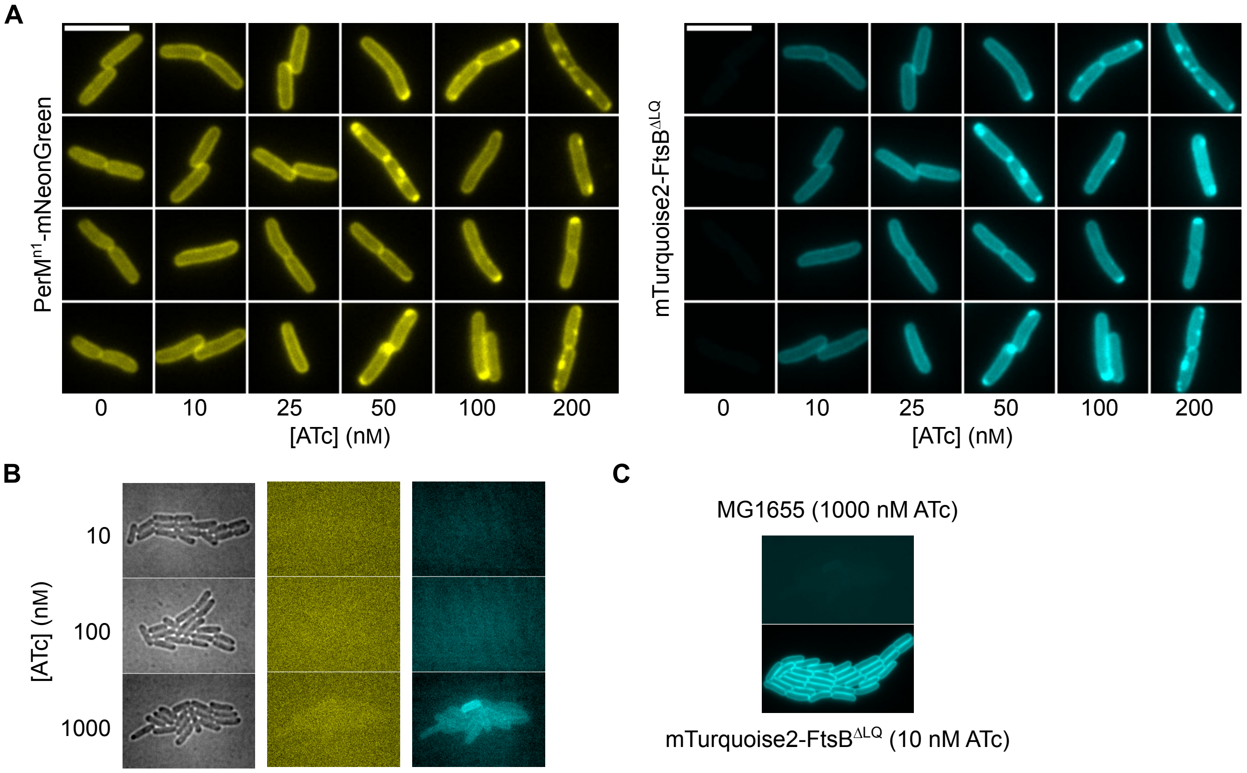

Fig. S3 ATc does not add substantial background to either mTurquoise2 or mNeonGreen images. (**A**) Cells with plasmids expressing PerM^{n1}-mNeonGreen and mTurquoise2-FtsB$^{\Delta LQ}$ were grown in the same conditions used for experiments reported in the main text with 100 µM IPTG and with ATc at concentrations up to 20×the maximum concentration used in analyzed data. All images for each channel are shown with the same minimum and maximum intensity scaling to facilitate comparison of background and cellular fluorescence intensity. ATc concentrations at and above 50 nM (5×the concentration used in our experiments) led to aggregation and a lack of further mTurquoise2 fluorescence increase outside of aggregates; aggregation of PerM^{n1}-mNeonGreen as well suggests that membrane protein translocation is beyond capacity for this strain and growth condition. No increase in background was observed in either channel at high ATc concentrations. Four regions of interest are shown for each condition with the same regions shown for both mNeonGreen and mTurquoise2 images. (**B**) MG1655 cells were grown in the same conditions used for experiments in the main text in the absence of antibiotics and the presence of different concentrations of ATc in growth media and agarose gel pad and imaged in brightfield as well as the same channels used for mTurquoise2 (cyan) and mNeonGreen (yellow) fluorescence imaging. The low contrast image shows that ATc-dependent cell fluorescence above background is evident only at an ATc concentration far above that used for PerM/FtsB imaging experiments. (**C**) The cyan image for MG1655 cells with 1000 nM induction is repeated from **B** and shown with identical minimum and maximum intensity scaling to a typical mTurquoise2-FtsB$^{\Delta LQ}$ microcolony image acquired using the same illumination and acquisition settings, but with 10 nM ATc.

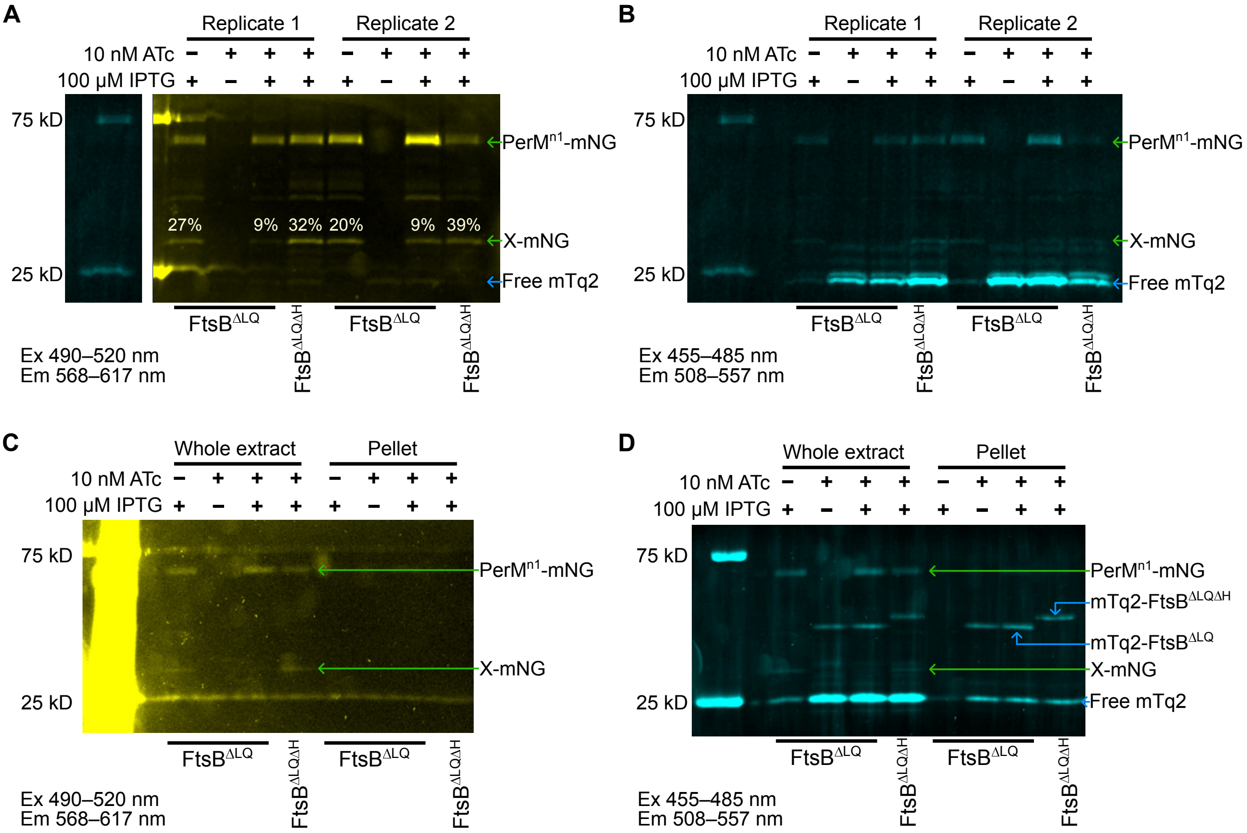

Fig. S4 Direct observation by fluorescent SDS-PAGE that PerM^{n1}-mNeonGreen degradation is reduced by interaction with FtsB via FtsB^{H}. (**A**) Cell extracts for strains that include plasmids expressing PerM^{n1}-mNG as well as mTq2-FtsB constructs with either FtsB$^{\Delta LQ}$ or FtsB$^{\Delta LQ\Delta H}$ were grown in different induction conditions and separated by SDS-PAGE. Extraction conditions were identified that maintained mNeonGreen and mTurquoise2 fluorescence, and solubilized full-length PerM^{n1}-mNG and its degradation products, but did not solubilize full-length mTq2-FtsB constructs well. Degradation of PerM^{n1}-mNG was quantified in two replicates of cell extracts as the integrated band intensity of the major degradation product (labeled X-mNG) relative to the sum of intensities for band X-mNG and the full-length PerM^{n1}-mNG band, which has a molecular weight approximately corresponding to that of PerM^{n1}-mNG (71 kD). Intermediate degradation products were not quantified. A weak band with lower molecular weight also appears in the absence of IPTG, and corresponds to a protein with mTurquoise2 fluorescence that is inefficiently detected in this channel. The ladder band is replaced with that from the blue/green image in **B** because its fluorescence signal was extremely high for this yellow/red filter set; intermediate bands in this ladder are not strongly fluorescent in either channel. Although both bright fluorescent bands give background in adjacent lanes, this did not significantly impact measuring the intensity of either PerM^{n1}-mNG band of interest. (**B**) The same gel as in **A** was imaged with a blue/green filter set. In the extraction conditions used to quantify PerM^{n1}-mNG degradation, full-length mTq2-FtsB constructs are not solubilized; instead, degradation products with mTurquoise2 fluorescence are observed near the expected 27 kD molecular weight of mTurquoise2. This is consistent with SDS-PAGE for other membrane protein fusions to fluorescent proteins [58]. Strong membrane localization for mTq2-FtsB$^{\Delta LQ}$ and mTq2-FtsB$^{\Delta LQ\Delta H}$ constructs in fluorescence microscopy also suggests that mTq2-FtsB proteolysis occurs during protein extraction, since free mTurquoise2 is expected to have cytoplasmic localization. (**C,D**) Another gel is imaged in the same channels as **A** and **B** and with the same growth and induction conditions. With modified extraction conditions (addition of a 30-minute incubation on ice), bands PerM^{n1}-mNG and fluorescent degradation products are poorly solubilized, while bands for mTq2-FtsB constructs are better solubilized. Identifiable bands are labeled and "X-mNG" signifies the unknown, fluorescent major degradation product of PerM^{n1}-mNG. Adding a centrifugation step results in a higher fraction of full-length mTq2-FtsB constructs in the resuspended pellet in **D**. Note the relative increase in the "X-mNG" band, indicating increased degradation of PerM^{n1}-mNG, in the absence of induction of mTq2-FtsB$^{\Delta LQ}$ and also in the absence of FtsB^{H}.
